# Reframing “flat affect” and withdrawal in severe mental illness: a within-subject, culture- and medication-sensitive heuristic for social psychiatry

**DOI:** 10.3389/fpsyt.2026.1717734

**Published:** 2026-03-11

**Authors:** Eik Niederlohmann

**Affiliations:** Department of Psychosomatic Medicine and Psychotherapy, Kliniken Erlabrunn, Breitenbrunn, Germany

**Keywords:** social psychiatry, severe mental illness, negative symptoms, flat affect, social withdrawal, within-person baseline, ICF-based functional assessment (Mini-ICF-APP), clinical decision support (PAD-S decision matrix)

## Abstract

Low facial expressivity and withdrawal in severe mental illness are often read as trait-like “flat affect” or enduring “negative symptoms”. In social-psychiatric and rehabilitation services, such labels can be pragmatically useful but clinically risky: they may narrow staff expectations, amplify stigma and self-stigma, and inadvertently shift the person’s narrative from participation and agency to deficit identity. This article proposes a service-level reframing: treat apparent flatness as a potentially state-dependent capacity signal that varies within-person across autonomic load, interpersonal context (including threat attribution to others), culture, and medication. Instead of anchoring interpretation in between-person norms, teams are encouraged to establish within-subject baselines (across contexts and time) and to document function-first impacts using International Classification of Functioning, Disability and Health (ICF)-aligned language. A simple three-zone pacing heuristic (Zones 1–3) is offered: proceed when regulated (Zone 1), reduce and structure interpersonal demand when tightening appears (Zone 2), and apply an explicit stop-and-ground pause when cognitive–perceptual disruption (CPD) emerges (Zone 3). The approach is complemented by a brief CPD screen [Cognitive-Perceptual Disruption Screening (COPEDS)] and copy-ready tools for co-regulation and documentation. This is a heuristic training aid, not a guideline and not primary evidence; it is intended to reduce misattribution risk, support continuity of care, and generate testable implementation questions for social psychiatry and psychiatric rehabilitation.

## 1 Introduction: why “flat affect” matters for social psychiatry

In rehabilitation-oriented services, “flat affect” is not a neutral descriptor: it can become a sticky trait label that shapes staff expectations, the person’s self-understanding, and the care pathway. Labels can support communication and access to care, yet they can also amplify stigma and self-stigma and narrow recovery-oriented goals (1–4).

This ambivalence is especially salient in social psychiatry and rehabilitation, where a diagnosis often functions as an administrative access code to services, benefits, or accommodations. When the label becomes reified as identity, however, it can carry downstream social costs (e.g., discrimination and insurance/employment barriers) and psychological costs (e.g., illness-identity consolidation, reduced agency, and learned helplessness). A service-level goal is therefore not “fewer diagnoses“ but better timing and better language: using diagnoses where needed for access and communication, while preventing premature trait attributions from colonizing the person’s recovery narrative (3, 4).

At the same time, low expressivity is a highly non-specific clinical sign. It can reflect negative symptoms assessed using established instruments, but it can also arise from state-dependent autonomic overload, context-specific threat appraisal, medication effects, or culturally patterned display norms (5–8). A classic lesson is that clinical interpretation is strongly context-sensitive: identical behavior can be read very differently depending on setting, role expectations, and institutional power (9). This article addresses a pragmatic service-level question: how can multidisciplinary teams avoid premature trait attribution when low expressivity and withdrawal appear in routine care?

Because “flat affect” is a non-specific clinical sign, a both/and biopsychosocial stance is useful: biological factors [acute medical/neurological change, medication, or extrapyramidal symptoms (EPSs)], psychological processes (e.g., anxiety and social-evaluative threat, shame, trauma-related threat appraisal, and depressive withdrawal), and social factors (authority dynamics, cultural display norms, and environmental stressors) can each contribute—and their relative prominence can shift over time. The present heuristic supports this stance by delaying trait attributions, translating context, pacing contact, and documenting function-first impacts while standard diagnostic work proceeds.

## 2 Scope and positioning (what this heuristic is and is not)

This article proposes a clinician-facing heuristic for routine social-psychiatric settings (wards, day programs, and community teams) where contacts are often brief, relationally intense, and shaped by structural constraints (time pressure, staffing ratios, coercion risk, and bureaucratic documentation).

The heuristic is not a diagnostic instrument or a treatment guideline, and it does not replace standard risk, diagnostic, or pharmacological assessment. Its purpose is pragmatic: to support safer interpretation and documentation in real time by translating observable patterns before trait attribution, pacing interpersonal demand, and protecting continuity of care through function-first notes.

**Figure 1:**
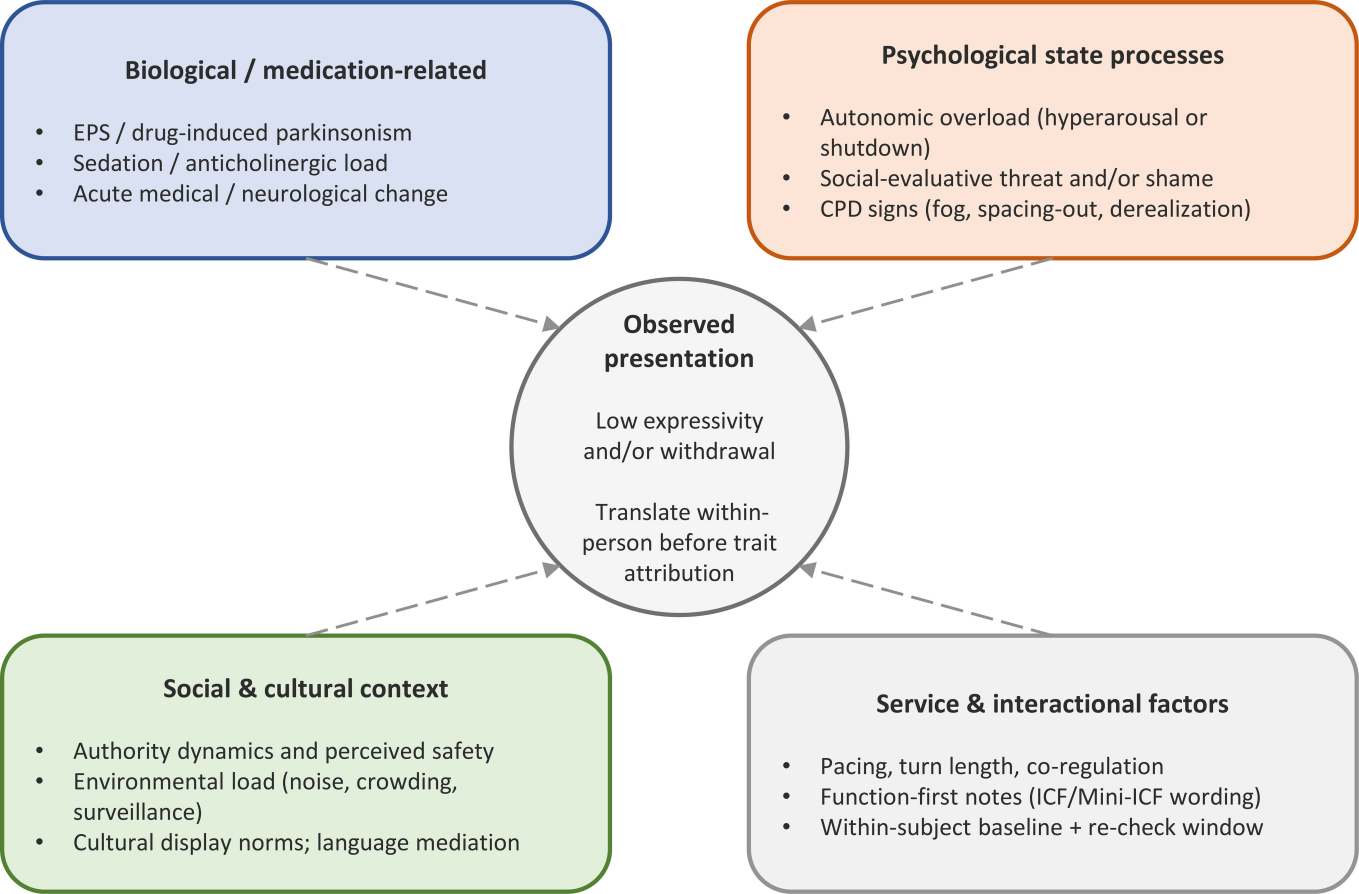
Ecological/biopsychosocial context map for interpreting low expressivity and withdrawal in routine social-psychiatric care. Low expressivity may reflect i) acute medical/neurological change and/or medication effects (including EPSs), ii) psychological overload (e.g., autonomic upshift, social-evaluative threat, shame, trauma-related threat appraisal, and depressive withdrawal), and/or iii) interpersonal, cultural, and service-level contexts (display norms, authority dynamics, coercion risk, and time pressure). The map is a structured prompt for team formulation and documentation, not a diagnostic instrument and not a deterministic causal model. Within-subject baselines and function-first notes (ICF/Mini-ICF) are emphasized; the Zone 1–3 pacing heuristic (**Figure 2**) provides one practical process tool during direct contacts. EPSs, extrapyramidal symptoms; ICF, International Classification of Functioning, Disability and Health; Mini-ICF, Mini-ICF Assessment for Psychological Disorders.

Conceptually, this paper distinguishes a process heuristic (a brief, safety-gated sequence for real-time pacing and documentation) from a system model (an exhaustive account of causes). The present contribution is the former; **Figure 1** is offered as an ecological prompt map (what to consider) and should not be read as a deterministic causal model of the person.

Practical prerequisites and boundary conditions:

Intended to be used alongside routine risk management and medication review; treat new or abrupt Zone 3/cognitive–perceptual disruption (CPD) signs as an acute-change signal that warrants standard medical/psychiatric assessment (e.g., sudden-onset hypomimia or other acute neurological change warrants standard medical workup).

Not designed as a stand-alone crisis protocol, nor as the sole basis for diagnosis, benefit decisions, or coercive measures.

Most useful when teams can document within-subject baselines and a re-check window (Section 5) and can access cultural/linguistic mediation when needed (Section 6).

Minimum requirements (plain language): i) staff can spend at least a few uninterrupted minutes in direct interaction; ii) the team has basic familiarity with Mini-International Classification of Functioning, Disability and Health (ICF) Assessment for Psychological Disorders (Mini-ICF-APP) domains (or an equivalent functional documentation tool) and can define a re-check window; and iii) there is a simple local agreement on who is authorized to pace contact (e.g., Zone 2/3 calls) and access to peer review/supervision for difficult cases.

Less suitable settings for standalone use: very high-turnover emergency departments with only seconds of contact, services working almost exclusively via indirect contacts, or systems with no infrastructure for basic functional documentation. In such contexts, treat the present framework only as a caution against premature trait attribution and default to standard acute assessment pathways.

Conceptually, the approach is compatible with a subsidiarity logic in rehabilitation: offer as much external scaffolding as needed to keep contact safe and collaborative, and return autonomy and initiative as soon as capacity increases. In this sense, the Zone 1–3 framing is intended as a shared team language for “how much interaction can this person bear right now, in this context?” rather than a fixed categorization of the person.

## 3 Reframing low expressivity as a within-subject capacity signal

A social-psychiatric reframing starts with the observation that expressivity varies strongly within the same person. Rather than comparing the person to group norms (“this looks flat”), the heuristic asks: how does this person look and sound at baseline, and how does that change as interpersonal demand rises (and in which contexts)?

When autonomic load increases, many people show a common threat-response cascade: facial and neck muscles tighten, blink rate drops, prosody narrows, and speech becomes clipped. In interpersonal contexts, threat appraisal can also become relationally amplified—fear is attributed to the other person (“they are interpreting/harming me”), which can further escalate autonomic load and yield a frozen, low-signal presentation that is easily misread as trait-like flatness. If load keeps rising, CPD can emerge (fog, spacing-out, and derealization) with a marked reduction in social signaling (10, 11).

Physiological models link these shifts to changes in arousal regulation, vagal tone, and interoceptive access (11). Social psychiatry adds two practical qualifiers: i) the same micro-phenotype can be elicited or prevented by context (e.g., authority-laden encounters, crowding, surveillance, and coercion risk) and ii) dyadic regulation is not optional—humans co-regulate, so staff behavior can either de-escalate or amplify the cascade. The still-face literature illustrates how quickly interactional “flatness” can emerge as a response to relational rupture, even in non-clinical dyads (12).

In experiential-dynamic traditions, similar shifts are described as anxiety flooding with protective muscle tension and (for some) a shutdown mode that protects against overwhelming affect. Outcome research suggests that emotion-focused, process-oriented work can be clinically useful across diagnostic categories, although the present paper does not claim efficacy for any specific intervention (13, 14).

Translated into social psychiatry, the implication is straightforward: apparent “flatness” can be a state marker of overload and reduced interactional capacity, not a stable deficit.

## 4 Zones 1–3: a pacing heuristic for real-world encounters

**Figure 2:**
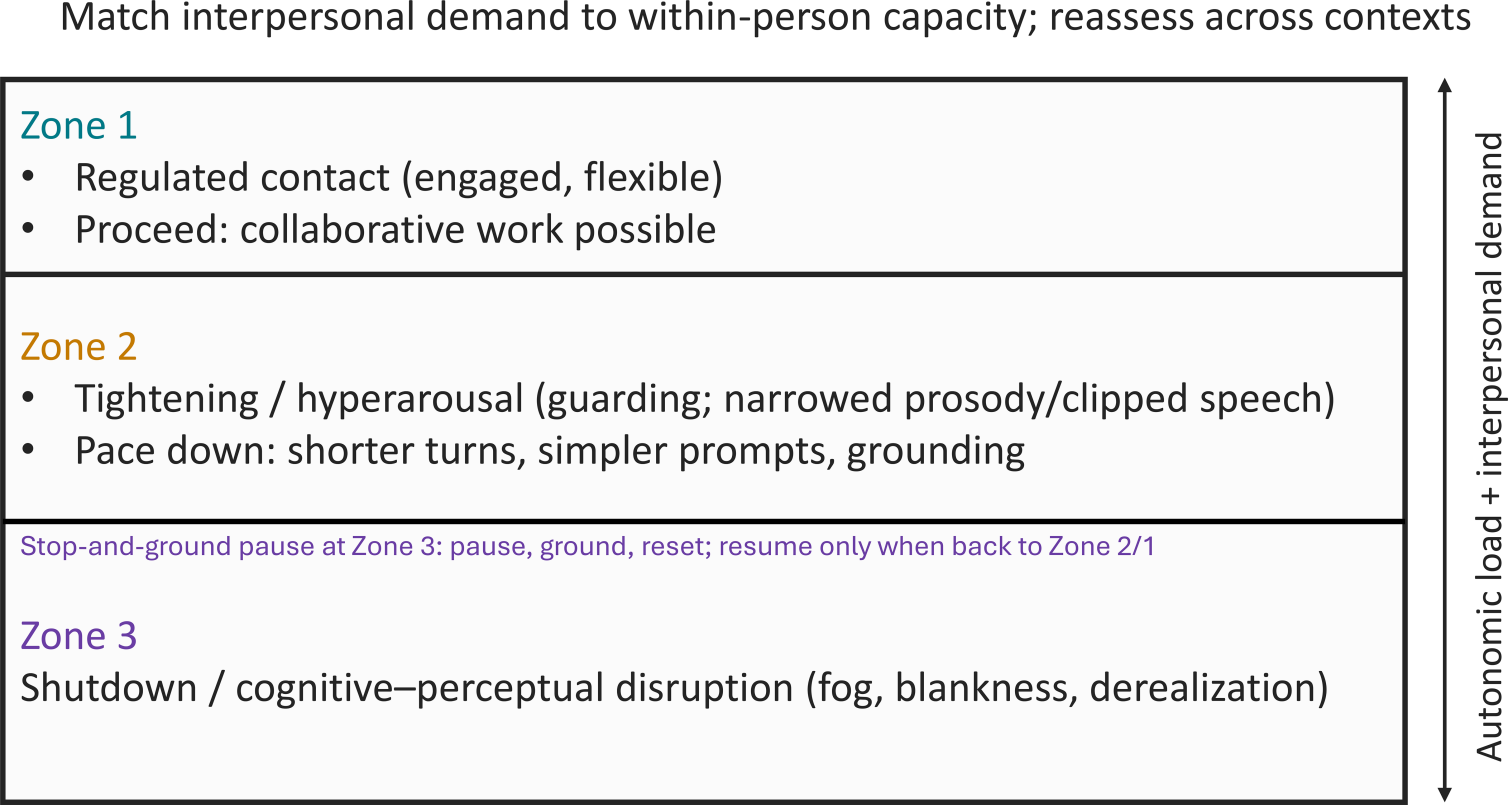
Zone 1–3 pacing model for within-person interpretation of low expressivity across contexts. The vertical axis indicates increasing autonomic load and interpersonal demand. Zone 1 (“regulated contact”) supports ordinary collaborative work (proceed). Zone 2 (“tightening/sympathetic upshift”) refers to guarding with narrowed prosody or clipped speech; pace down by reducing and structuring interpersonal demand (shorter turns, simpler prompts, and grounding). Zone 3 indicates shutdown with cognitive–perceptual disruption (CPD; fog, blankness, and derealization) and triggers an explicit stop-and-ground pause (pause, ground, and reset); resume only when the person returns to Zone 2 or Zone 1. Heuristic training aid (no primary data); to be used alongside standard diagnostic, risk, and pharmacological assessment procedures; not a guideline and not a standalone decision tool.

**Figure 2** summarizes a three-zone pacing model for within-person contact. The zones are descriptive, not diagnostic; they support team agreement about what to do next.

Zone 1 (regulated): the person is engaged enough for collaborative work; proceed in ordinary turns.

Zone 2 (tightening/hyperarousal): protective muscle tension with narrowed prosody or clipped speech (and/or withdrawal) appears as demand rises; reduce interpersonal load (shorter turns, simpler prompts, and grounding).

Zone 3 (shutdown/CPD): fog, blankness, disorientation, or derealization emerges; apply a stop-and-ground pause and resume only when the person returns to Zone 2 or 1.

**Supplementary Material S1** provides a one-page quick reference, and **Supplementary Material S2** offers a 60-second co-regulation script for Zone 2/3 encounters.

A practical advantage of this framing is psychoeducation: staff can explain the shift as “nervous system overload under social demand” rather than as a fixed deficit and can offer a concrete, collaborative pathway (pace → co-regulate → graded exposure) (4).

This is also where social-psychiatric and interpersonal perspectives meet. Empathy, operationally, includes calibrating the form and intensity of contact to what the other person can bear; a “warmer” or more demanding style may be supportive for one person but invasive for another (15). At the service level, the same logic functions as subsidiarity: scaffold just enough to keep participation possible and then hand initiative back.

In an active inference framing, rehabilitation continually balances “perception: change beliefs” and “action: change world” (16). Zones 1–3 can be read as a micro-level tool at this interface: in Zone 2/3, teams may need to change the immediate interactional environment (action: reduce interpersonal load) so that the person can update threat appraisals and re-engage (perception: regain access to signals).

## 5 Documentation: function-first language for continuity of care

Social psychiatry depends on continuity: different staff, settings, and timepoints must be able to read each other’s notes without importing stigma or moral interpretation. A function-first approach supports this by documenting observable participation and capacity impacts rather than decontextualized trait labels (17).

This matters politically as well as clinically: even when diagnostic labels are required for administrative access, how teams write can either reinforce a fixed deficit narrative (“this person is flat/unmotivated”) or keep the focus on modifiable barriers and supports (“under high dyadic load, initiation drops; tolerates brief turns; improves with grounding”).

Mini-ICF-APP tools operationalize this interface for mental disorders and can help teams record what changes under load (endurance, interaction, and planning/structuring) and what helps (pacing, grounding, and graded exposure) (18). **Supplementary Material S3** provides an expanded Mini-ICF-APP crosswalk for implementation teams. For compactness, the cue → translation → response templates and example one-line Mini-ICF sentences are provided as **Supplementary Material S4**. **Supplementary Material S1** also provides two brief micro-examples showing how structurally patterned expectations (e.g., eye-contact norms under evaluation) can distort interpretation and how within-subject translation can reduce harm.

## 6 Culture and medication safeguards

Culture enters twice: as display norms (what counts as “appropriate” expressivity) and as meaning (what silence, withdrawal, or emotional restraint signify in a local moral and social order). In some contexts, restrained affect is normative; in others, expressing distress may be stigmatized or gendered. A culture-sensitive safeguard is therefore to compare within-person baselines across contexts (in-group vs. out-group; private vs. formal encounters) and to use cultural consultation or mediation where needed (7, 8, 19).

Medication enters in parallel. Extrapyramidal symptoms and drug-induced parkinsonism can reduce blink rate and facial/vocal expressivity and may mimic “flatness” (20). Sedating or anxiolytic agents can directly change emotional processing (21), and selective serotonin reuptake inhibitors (SSRIs) can alter reinforcement sensitivity (22). Opioids can modulate social signaling, including facial mimicry and emotion recognition (23, 24).

These influences are not “confounds” to be ignored: in real-world rehabilitation, they are part of the ecology. The practical safeguard is to avoid trait attribution until i) within-subject baselines have been established across contexts and time and ii) medication effects (including EPS/sedation) have been considered.

**Figure 1** summarizes an ecological/biopsychosocial context map for reading low expressivity and withdrawal in routine services. It is intended as a shared checklist of plausible contributors (biology/medication, psychological overload, interpersonal context, culture, and service ecology) that helps teams delay trait attribution, establish within-subject baselines, and select proportionate pacing and documentation responses.

## 7 CPD screening and negative symptom constructs: a both/and approach

CPD matters clinically because it changes what is possible in the encounter: when fog, derealization, or disorientation is present, deeper exploration and higher interpersonal demand can become iatrogenic. For teams who want a brief screen, Cognitive-Perceptual Disruption Screening (COPEDS) (a short CPD screening instrument developed in outpatient psychotherapy settings) may help triage when a “stop-and-ground” approach is indicated (25). Because COPEDS was not developed for acute medical syndromes, intoxication/delirium, or psychosis-spectrum states, it should be treated as a safety prompt (slow down, ground, and re-orient) rather than a diagnostic instrument.

Outside its validation context, COPEDS scores should be read cautiously. In more complex populations (e.g., prominent psychosis-spectrum experiences, heavy substance use, neurodevelopmental conditions, or markedly different cultural idioms of distress), items may plausibly yield false positives (endorsed for other reasons) or false negatives (under-reporting of fog/derealization). In such groups, treat COPEDS as hypothesis-generating and prioritize standard clinical assessment and collateral information.

Minimum response to a positive COPEDS screen or clear CPD signs in routine services (even without a specialized graded format): i) avoid prolonged high-load interpersonal exposure without monitoring for CPD signs; ii) use a brief, predefined check of who is authorized to decide on paced versus standard contact; and iii) document CPD/recovery windows and helpful supports in Mini-ICF language so that other staff can reproduce the safer format across shifts.

Importantly, this article does not deny the clinical reality of negative symptoms. Instead, it targets a common service error: treating low expressivity as automatically trait-like. Standard negative symptom instruments (e.g., BNSS and CAINS) remain appropriate for structured assessment (5, 6); the proposal here is a pragmatic complement for routine contacts—before formal ratings are available and before teams harden a narrative. For example, before rating blunted affect as a persistent trait on a negative symptom scale, teams are encouraged to consider Zone 1–3 patterns across contexts and medication status.

In practice, teams can hold both frames: i) a longitudinal negative symptom formulation where indicated and ii) a moment-to-moment capacity formulation that asks whether the current presentation may reflect overload, medication effects, cultural restraint, or CPD. This “both/and” stance can reduce stigma while preserving diagnostic precision.

## 8 Implementation priorities and testable questions

Implementation can be low-cost: brief staff teaching on Zones 1–3, shared language (“tightening”, “stop-and-ground”, and “protect positives”), and one-line Mini-ICF notes. The key is fidelity to the translation step: observable cue → hypothesized mechanism (overload/meds/culture) → proportionate response.

A social-psychiatric risk is drifting into chronic over-helping (which can undermine agency) or chronic under-helping (which can amplify crises). Using the zones as a subsidiarity prompt (“How much scaffolding is needed right now?”) may help teams step down support as capacity increases and step up early when CPD signs appear.

At a broader level, rehabilitation is rarely an either/or between “change the person” and “change the world”; a both/and biopsychosocial stance is often required. Short-term, social interventions often need to change the world (reduce stressors, secure housing/benefits, and provide structured daily activity); longer-term, many people also benefit from updating beliefs, attachment expectations, and self-concepts so that agency and participation become sustainable (16).

Two brief examples illustrate the logic. After a painful separation, acute supportive measures (reduced demands, practical help, and containment) may be essential; later, a person may still need to work on recurring relational patterns that make similar crises more likely. In psychosis, maladaptive belief updating can produce a subjectively coherent “solution” (e.g., a fixed relationship belief) that drives actions that further damage social ties; in such cases, environmental support and reality-based relational coaching may be needed alongside careful work on threat appraisals and affect tolerance.

Testable service-level questions include the following: Does training in the heuristic reduce trait-language in charts? Does it improve alliance and perceived respect? Does Mini-ICF documentation show improved participation at follow-up? Crucially, for stigma, does within-subject, state-sensitive framing reduce self-stigma proxies and diagnostic overshadowing in routine decisions?

## 9 Discussion and limitations

The proposed heuristic is intentionally modest: it does not redefine negative symptoms, and it does not claim that “flat affect” is always anxiety-related. It offers a translation habit for busy teams: treat low expressivity as a potentially state-dependent capacity signal until proven otherwise, and intervene proportionately by pacing contact, protecting safety, and documenting function-first effects.

This framing aligns with a core tension in social psychiatry: diagnoses are sometimes necessary as “entry tickets” to support systems, yet they can also carry stigma, structural penalties, and identity effects. Because health and welfare systems differ internationally, the same label can function as protection in one context and as social liability in another. A within-subject, function-first approach can help teams preserve the administrative utility of diagnosis while reducing the risk that a diagnosis becomes the person’s total story (3, 4).

Evidence boundaries should be explicit. The strongest empirical components are the existing measurement and documentation tools (BNSS/CAINS for negative symptoms, Mini-ICF-APP for functioning, and COPEDS for CPD screening) and the medication effects literature (5, 6, 18, 20–25). By contrast, the Zone 1–3 pacing model and the specific “stop-and-ground” routine are heuristic extrapolations intended for training and local quality improvement; they should be treated as testable service routines rather than established facts.

Briefly, several adjacent literatures converge on the same practical point: symptoms and participation are enacted in social worlds and shift with context. Phenomenological/enactive work emphasizes meaning and normativity, systemic therapy emphasizes a “both/and” stance across explanatory levels, and computational psychiatry is beginning to express clinical micro-decisions in structured state–action formats that could help evaluate or digitally support simple pacing routines such as Zones 1–3. These pointers are included only as an optional outlook; the heuristic does not depend on them (26–29).
