# Supplementary material for: Reframing “flat affect” and withdrawal in severe mental illness: a within-subject, culture- and medication-sensitive heuristic for social psychiatry
Source: Front Psychiatry. 2026 Mar 11;17:1717734. doi: 10.3389/fpsyt.2026.1717734 (PMC13013531; doi:10.3389/fpsyt.2026.1717734)
Supplement: Supplementary file 1 [file DataSheet1.pdf]

Supplementary Material S1. Heuristic steps and stop-and-ground pause (quick reference)

Scope: bedside heuristics to support within-subject, culture- and medication-sensitive reading of low expressivity in routine social-psychiatric care.

Heuristic training aid - no primary data. Not a guideline. Use only alongside standard diagnostic, risk, and pharmacological assessment procedures; not as a standalone decision tool.

Intended use: Training aid corresponding to Sections 4–7 of the main text.

- Translate before trait attribution: describe the observable pattern + context first; avoid trait labels.
- Process heuristic (pacing + documentation) rather than system model: use as a real-time aid, not as a deterministic decision protocol or a description of the person.
- Both/and biopsychosocial check (plain language): before trait attribution, consider whether low expressivity reflects (i) acute medical/neurological change and/or medication/EPS, (ii) psychological overload (e.g., anxiety, social-evaluative threat, shame), and/or (iii) social context and cultural display norms. Reassess over time with within-subject baselines.
- Pace contact by Zones 1–3: Zone 1 = proceed; Zone 2 = pace (shorter turns, simpler prompts, more grounding); Zone 3 = stop and stabilize until back to Zone 2/1.
- 60-second co-regulation plan (Zone 2 or early Zone 3): orient to present safety; externalize self-attack; offer one concrete prompt in ~5-10 s windows; check gaze/body re-engagement; repeat as needed.
- Explicit stop-and-ground pause at Zone 3 (CPD): at fogging, spacing-out, disorientation, or derealization - pause, ground, and reset; resume only when back to Zone 2/1.
- Write one Mini-ICF sentence (function-first): a brief functional note for teams (context, zone, pacing action, re-check window).

Micro-examples (structural misinterpretation; for training):

• Authority-laden round / group meeting: reduced eye contact and minimal prosody may be misinterpreted (e.g., read as ‘uncooperative’) or filtered through culturally and racially patterned expectations. Translate first (context + within-subject baseline), then pace contact (Zone 2) and verify engagement in a calmer 1:1 context before trait attribution.

• Gendered/class-linked readings: silence, reserve, or tearfulness may be interpreted through local norms rather than capacity. Use the translation step (overload vs. restraint norms), reduce interpersonal demand, and document function-first impacts (Mini-ICF) so that other staff do not escalate pressure.

After a positive COPEDS screen or clear CPD signs: avoid prolonged high-load exposure without monitoring; clarify who is authorised to pace contact; document CPD/recovery windows in one Mini-ICF sentence for continuity.

Abbreviations: CPD = cognitive-perceptual disruption; Mini-ICF = Mini-ICF-APP. See Supplementary Material S3 for Mini-ICF examples.
