# Supplementary material for: Reframing “flat affect” and withdrawal in severe mental illness: a within-subject, culture- and medication-sensitive heuristic for social psychiatry
Source: Front Psychiatry. 2026 Mar 11;17:1717734. doi: 10.3389/fpsyt.2026.1717734 (PMC13013531; doi:10.3389/fpsyt.2026.1717734)
Supplement: Supplementary file 2 [file DataSheet2.pdf]

Supplementary Material S2. 60-second co-regulation script

Heuristic training aid - no primary data. Not a guideline. Use only alongside standard diagnostic, risk, and pharmacological assessment procedures; not as a standalone decision tool.

Intended use: Training aid corresponding to Sections 4 and 8 of the main text.

Two-column script for families/peers and clinicians. Adapt wording to the person's language and cultural norms.

**Plain language (person/family)**	**Clinician prompts/cues**
Let’s pause. Feel your feet and the chair; we’re safe here.	Orient to present safety; slow breath/grounding.
That harsh inner voice is just a protector; it’s not the truth.	Name and externalize harsh inner critic/self-attack.
We’ll take this in very small steps.	Offer single concrete prompt; keep about 5-10 s windows.
Tell me one short sentence; then we pause again.	Contain exposure; monitor gaze/tone; adjust Zones 1–3.
If your mind goes blank, we stop and reset together.	Apply stop-and-ground pause at CPD; resume only when back to Zone 2/1.

Tip: read out loud at natural pace; keep silences. Check orientation and consent continuously.
