# Supplementary material for: Reframing “flat affect” and withdrawal in severe mental illness: a within-subject, culture- and medication-sensitive heuristic for social psychiatry
Source: Front Psychiatry. 2026 Mar 11;17:1717734. doi: 10.3389/fpsyt.2026.1717734 (PMC13013531; doi:10.3389/fpsyt.2026.1717734)
Supplement: Supplementary file 3 [file DataSheet3.pdf]

Supplementary Material S3. Mini-ICF-APP crosswalk for functional documentation

Use these anchors to replace trait labels with activity/participation impacts that travel across settings.

Heuristic training aid - no primary data. Not a guideline. Use only alongside standard diagnostic, risk, and pharmacological assessment procedures; not as a standalone decision tool.

Intended use: Documentation aid corresponding to Section 5 of the main text.

Observation (clinical)	Mini-ICF-APP domain	One-line wording (example)
Tightening under task request	Planning/structuring; Endurance	Under dyadic load: reduced endurance, needs short, concrete prompts
Praise -> self-attack	Interpersonal interactions	Protect positive affect; validation unlocks participation
Conflict -> gaze down, minimal prosody	Contacts/interactions	Participates in calm 1:1; graded exposure in conflict
CPD signs (fog/blank)	Adapting to demands	Momentary disorganization; benefits from pause/grounding
Sedated tone/mask-like facies	Motor skills/energy	Motor expressivity reduced under meds; function intact at rest
Family meeting oscillation Zone 2→3	Contacts/interactions; Endurance	Fluctuating relatedness; tolerates brief, paced turns
Authority-laden group setting -> reduced eye contact / speech	Contacts/interactions; Endurance	In group rounds: participation reduced; in calm 1:1: engages; plan graded exposure; consider cultural mediator
COPEDS positive + fog under load	Adapting to demands; Planning/structuring	Under dyadic load: transient disorganization; benefits from grounding and shorter turns; re-check after capacity building

Mini-ICF-APP domains are examples; adapt to local documentation requirements.
