# Supplementary material for: Reframing “flat affect” and withdrawal in severe mental illness: a within-subject, culture- and medication-sensitive heuristic for social psychiatry
Source: Front Psychiatry. 2026 Mar 11;17:1717734. doi: 10.3389/fpsyt.2026.1717734 (PMC13013531; doi:10.3389/fpsyt.2026.1717734)
Supplement: Supplementary file 4 [file DataSheet4.pdf]

Supplementary Material S4. Cue → translation → response templates with Mini-ICF sentence examples

Scope: documentation templates to support within-subject, culture- and medication-sensitive reading of low expressivity in routine social-psychiatric care.

Heuristic training aid - no primary data. Not a guideline. Use only alongside standard diagnostic, risk, and pharmacological assessment procedures; not as a standalone decision tool.

Intended use: Documentation aid corresponding to Section 5 (Documentation) of the main text.

See Figure 2 (main text) for an ecological/biopsychosocial context map that can be used alongside these templates.

Note: Templates are examples for training and team calibration, not prescriptive instructions. Use within-subject baselines and reassess over time; avoid trait attribution until medication and cultural factors have been considered.

Observable cue	Mechanism (translation)	Suggested immediate response	Mini-ICF sentence (example)
Task request → expression tightens, clipped speech	Probable overload (ANS upshift)	Consider Zone 2 pacing: shorter turns, simpler prompts, grounding	Under dyadic load, reduced initiation; tolerates about 5–10 s turns; re-check in 6–8 weeks
Praise → sudden stillness / self-attack	Shame spike; positive affect triggers harsh self-criticism	Protect positives; externalize self-attack; brief, low-demand contact	Fluctuating relatedness with positives; needs validation then brief prompts
Disagreement → gaze down, minimal prosody	Threat appraisal; cultural restraint possible	Translate before labeling; check culture/meds; keep windows brief	Participation consistent in calm 1:1; reduced in conflict; plan graded exposure
Rising blankness / “fog”	Cognitive–perceptual disruption (CPD)	At Zone 3, pause: name overload, ground, resume at Zone 2/1	Momentary disorganization under load; benefits from pause/grounding

Sedated tone; reduced blink; mask-like facies	Medication effect/EPS more likely than trait	Consider medication review; screen for EPS before labeling	Motor expressivity reduced under current meds; function intact at rest
Family meeting → oscillation Zone 2→3	Attachment-laden triggers exceed window	Pace: brief co-regulation; protect positives; consider shorter segments	Dyadic relatedness fluctuates; graded exposure; re-check in 6–8 weeks
Authority round / group meeting → reduced eye contact, minimal prosody	Norm-congruent restraint OR threat under evaluation (risk of structural bias)	Translate before labeling; verify within-subject baseline in safer contexts; prefer calm 1:1; consider cultural consultation/mediator	In authority-laden groups: participation reduced; in 1:1 safe context: engages with prompts; plan graded re-exposure; re-check in 6–8 weeks
Misread as "unwilling" → staff increases pressure → blankness increases	Iatrogenic overload spiral (pacing mismatch)	Step back to Zone 2: reduce interpersonal load; apply an early stop-and-ground cue at CPD signs; document pacing mismatch to prevent repetition	Under excess demand: Zone 2→3 drift; needs shorter turns and grounding; participation improves when pacing is reduced; re-check in 4–6 weeks

Abbreviations: ANS = autonomic nervous system; CPD = cognitive–perceptual disruption; EPS = extrapyramidal symptoms; Mini-ICF = Mini-ICF-APP.
